# Zebra-Sphinx: Modeling Sphingolipidoses in Zebrafish

**DOI:** 10.3390/ijms24054747

**Published:** 2023-03-01

**Authors:** Luca Mignani, Jessica Guerra, Marzia Corli, Davide Capoferri, Marco Presta

**Affiliations:** Unit of Experimental Oncology and Immunology and Zebrafish Facility, Department of Molecular and Translational Medicine, University of Brescia, 25123 Brescia, Italy

**Keywords:** gene knockout, hereditary disease, lysosome, morpholino, sphingolipid, zebrafish

## Abstract

Sphingolipidoses are inborn errors of metabolism due to the pathogenic mutation of genes that encode for lysosomal enzymes, transporters, or enzyme cofactors that participate in the sphingolipid catabolism. They represent a subgroup of lysosomal storage diseases characterized by the gradual lysosomal accumulation of the substrate(s) of the defective proteins. The clinical presentation of patients affected by sphingolipid storage disorders ranges from a mild progression for some juvenile- or adult-onset forms to severe/fatal infantile forms. Despite significant therapeutic achievements, novel strategies are required at basic, clinical, and translational levels to improve patient outcomes. On these bases, the development of in vivo models is crucial for a better understanding of the pathogenesis of sphingolipidoses and for the development of efficacious therapeutic strategies. The teleost zebrafish (*Danio rerio*) has emerged as a useful platform to model several human genetic diseases owing to the high grade of genome conservation between human and zebrafish, combined with precise genome editing and the ease of manipulation. In addition, lipidomic studies have allowed the identification in zebrafish of all of the main classes of lipids present in mammals, supporting the possibility to model diseases of the lipidic metabolism in this animal species with the advantage of using mammalian lipid databases for data processing. This review highlights the use of zebrafish as an innovative model system to gain novel insights into the pathogenesis of sphingolipidoses, with possible implications for the identification of more efficacious therapeutic approaches.

## 1. Introduction

**Figure 1:**
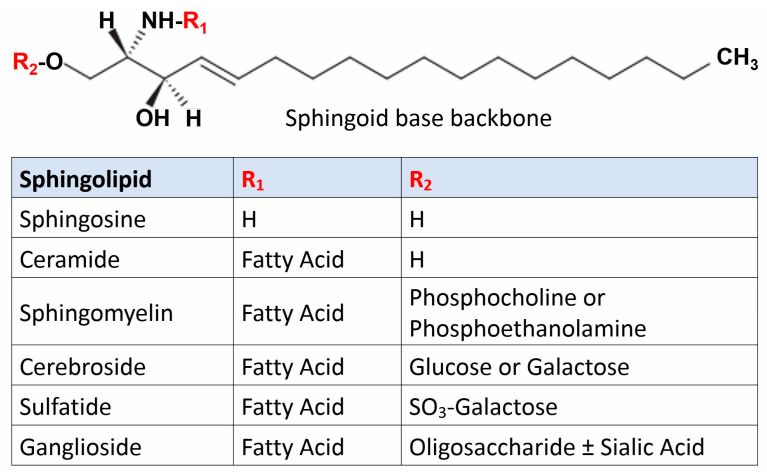
Schematic structure of sphingolipids.

Sphingolipids were first described during the second half of the nineteenth century [1]. The term “sphingolipid” was coined based on the complexity and sphinxlike nature of this class of lipids characterized by a core long chain aliphatic amino alcohol (sphingoid base). The most common member is represented by sphingosine, which can be functionalized by a fatty acid condensed at its aminic moiety and by polar molecules at its hydroxyl terminus, including small organic molecules, amino acids, or carbohydrates [2] (Figure 1).

De novo synthesis of sphingolipids begins in the endoplasmic reticulum (ER) and may move towards the Golgi apparatus. Finally, their mature forms are delivered to cell membranes [2,3,4]. Sphingolipids play a key structural role in cellular membranes and/or act as signaling molecules. Owing to their molecular structure, sphingolipids can organize within plasma membranes into ordered focal regions named lipid rafts, crucial for the arrangement of raftophilic molecules or transmembrane protein domains [5]. During recycling or after signaling events, sphingolipids may reach the lysosomes, where specific enzymes catabolize them to less-complex molecules, which can enter different metabolic pathways or act as novel signaling molecules [6]. Educated reviews have described the anabolic and metabolic pathways that characterize the sphingolipid metabolism and the involvement of the different sphingolipid species in physiological and pathological processes [7,8].

**Figure 2:**
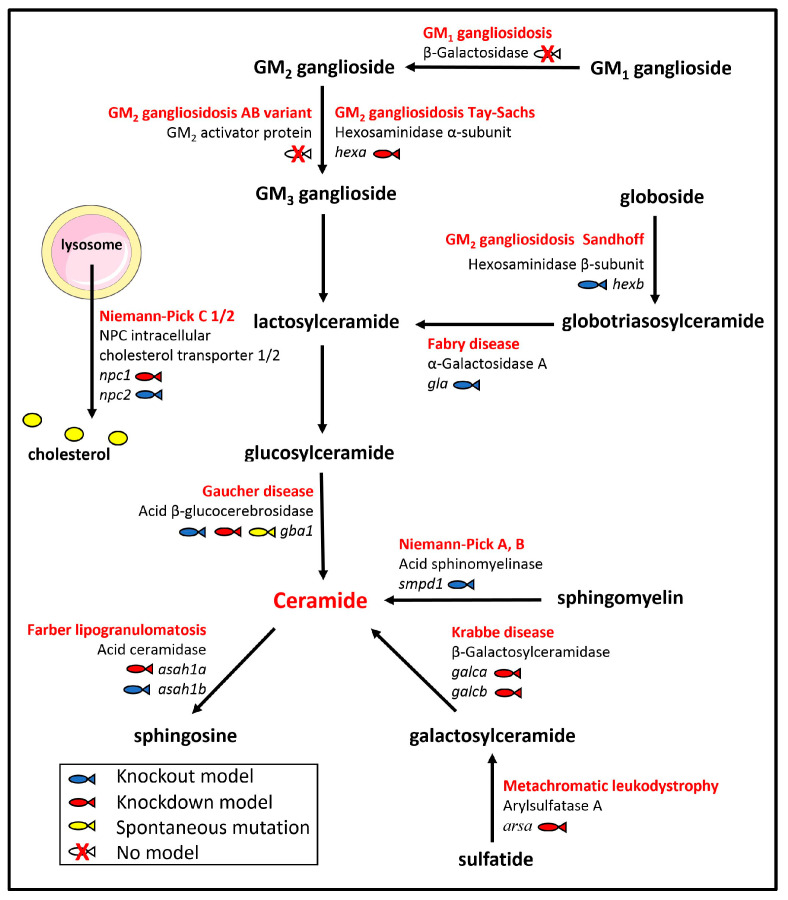
Sphingolipid catabolism. Schematic representation of the biochemical pathways of the sphingolipid catabolism related to hereditary human sphingolipidoses and corresponding non-functional enzymes. Zebrafish genes that have been knocked out or knocked down to generate models of sphingolipidoses are in italics. See text for details.

Lysosomal storage diseases are a group of genetic disorders characterized by the gradual lysosomal accumulation of metabolites due to a defective lysosomal hydrolytic activity [9]. Among them, alterations of the lysosomal catabolic pathways responsible for the progressive breakdown of complex sphingolipids may translate into the accumulation of their corresponding undegraded substrates in lysosomes, leading to inherited sphingolipid storage diseases gathered under the name of sphingolipidoses [10]. In this review, we will focus our attention on zebrafish (*Danio rerio*) as an animal model for the study of this sub-class of lysosomal storage diseases and as a “zebra-sphinx” platform for a better understanding of the complex and, at least in part, still sphinxlike biology of sphingolipid metabolism (Figure 2).

## 2. Sphingolipidoses

Sphingolipidoses affect approximately 1 in 20,000 newborns [11]. The clinical presentation of patients affected by sphingolipid storage disorders is quite diverse, ranging from a mild progression for some juvenile- or adult-onset forms to severe and fatal infantile forms.

**Figure 3:**
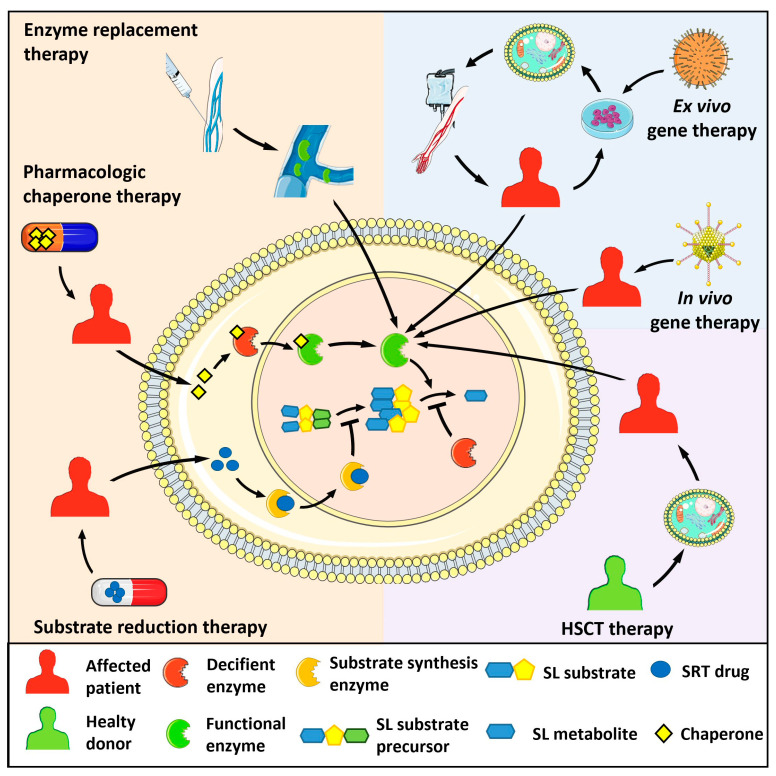
Therapeutic strategies for the treatment of sphingolipidoses. Enzyme replacement therapy consists of the intravenous administration of a bioactive recombinant form of the deficient enzyme. Pharmacologic chaperon therapy favours the proper folding of the mutated, misfolded enzyme and its lysosomal translocation, allowing the hydrolysis of the engulfing sphingolipid (SL) substrate. In substrate reduction therapy (SRT), drugs inhibit the activity of the enzyme responsible for the synthesis of the SL substrate of the deficient enzyme, hampering its lysosomal accumulation. In hematopoietic stem cell transplantation (HSCT), healthy donor-derived cells provide the patient with cells expressing the functional enzyme. Ex vivo gene therapy administers a bioactive enzyme by autologous transplantation of genetically modified hematopoietic stem cells. In vivo gene therapy consists of the injection of viral vectors encoding for the functional enzyme.

To date, approved and investigational therapies for the treatment of lysosomal storage diseases, including sphingolipidoses, comprise hematopoietic stem cell transplantation (HSCT), in vivo and ex vivo gene therapy, enzyme replacement therapy (ERT), substrate reduction therapy (SRT), and pharmacologic chaperone therapy [12,13,14] (Figure 3). These strategies have improved the life of many affected patients by preventing progression or ameliorating various signs and symptoms. However, given the complexities resulting from the alterations of sphingolipid metabolism in different systemic organs, much is still needed at the basic, clinical, and translational levels to improve patient outcomes. For the purposes of the present paper, we will briefly describe the major types of human sphingolipidoses (Table 1). Diseases associated with deficiency of the sphingolipid activator proteins saposins A-D generated by proteolytic processing of the common precursor prosaposin will not be described here.

### 2.1. Gaucher Disease

Gaucher disease (GD) is one of the most common sphingolipidoses with an incidence ranging from 1:40,000 to 1:60,000 live births in the general population, with 1:850 in the Ashkenazi Jewish population [15]. GD is caused by recessive mutations in the *GBA1* gene that encodes for acid β-glucocerebrosidase, also known as β-glucosidase, a lysosomal enzyme responsible for the degradation of glucosylceramide.

The deficiency of acid β-glucocerebrosidase activity leads to the accumulation of its substrate primarily in the lysosomes of macrophages (Gaucher cells) found in the spleen, liver, bone marrow, lungs, and lymph nodes of affected patients [16].

Marked enlarged liver and splenomegaly are clear signs of the disease in children and teenagers that give rise to defects in the blood circulation with anemia and bleeding tendency [17]. Gene expression analysis of cultured skin fibroblasts from GD patients demonstrated that glucosylceramide accumulation triggers the activation of inflammatory responses via the upregulation of genes involved in cytokine and JAK-STAT signaling pathways, the downregulation of genes involved in cell-to-cell and cell-to-matrix interaction, and the inhibition of PI3K-Akt and survival signaling pathways [18].

Several factors may contribute to the severity of GD depending on the type of *GBA1* mutation, including the levels of ER stress and proteasomal degradation. In particular, ER stress responses may entail the accumulation of α-synuclein aggregates, causative of neuronal injury and degeneration, as in Parkinson’s disease [19,20]. 

According to the degree of severity and impairment, GD is classified into three main groups (GD type I–III) based on clinical presentation. The most frequent and less aggressive form of GD is type I, also known as nonneuropathic GD. The onset of the disease varies from childhood to adulthood, and is characterized by bone pain and fractures, splenomegaly, hepatomegaly, anemia, leukopenia, and thrombocytopenia [17]. Although it is considered nonneuropathic, a continuum of clinical forms between GD types may exist, with some neuropathic defects also observed in GD type I patients [21].

GD type II and GD type III are historically classified as primary neurologic diseases. GD type II represents the most severe form as it affects children eliciting rapid degeneration that leads to death before 4 years of age. GD type III usually has a later onset with slower progression [22]. 

Nowadays, macrophage-directed ERT is the standard of care for symptomatic GD type I and type III patients. It is efficacious in reducing splenomegaly and hematological signs, favoring the growth of GD children, whereas, at variance with ERT, SRT based on the administration of glucosylceramide synthase inhibitors has been shown to be effective in also reducing the skeletal complications [23]. At present, no approved treatment exists for neuropathic GD, but recent studies suggest that the use of ambroxol, an over-the-counter drug that can cross the blood–brain barrier, might be effective [24].

### 2.2. Fabry Disease

The Fabry disease, also known as the Anderson–Fabry disease, was first described by W. Anderson and J. Fabry in 1898 as a systemic vascular disorder [25]. The Fabry disease is a X-linked disorder caused by mutations in the *GLA* gene encoding for α-galactosidase A that catalyzes the hydrolysis of terminal non-reducing α-d-galactose residues in α-D-galactosides [26]. 

Pathogenic variants in *GLA* result in absent or non-functional α-galactosidase A, leading to the accumulation of its substrate globotriaosylceramide (Gb3) and the deacylated derivative globotriaosylsphingosine in the lysosomes of endothelial cells, myocytes, renal cells, and neurons [27,28]. 

At the molecular level, the pathogenesis of Fabry disease is still unclear [29]. Gb3 accumulation results in the deregulation of the mitochondrial function and of mTOR and autophagy/lysosome pathways in peripheral blood mononuclear cells from Fabry patients. Of note, similar lysosomal, autophagy, and mitochondrial alterations were also observed in Faber cells, suggesting that a common pathogenic mechanism may exist for both sphingolipidoses [30]. Further confirmation that autophagy and mitochondrial dysfunctions may occur in Fabry disease comes from studies performed on cardiovascular endothelial cells derived from Fabry-induced pluripotent stem cells in which the *GLA* mutation was corrected by clustered regularly interspersed short palindromic repeats/CRISPR-associated 9 (CRISPR/Cas9) technology [31].

The Fabry disease is typically divided into the major classical or infantile phenotype and the late-onset phenotype. The classical form of Fabry disease affects males that have little or no residual α-galactosidase A activity. It is characterized by clinical heterogeneity and symptoms arise around 1 to 3 years of age. Children with classical Fabry disease usually present acroparesthesia (“Fabry crisis”), angiokeratoma, hypohidrosis, and heat intolerance. The initial symptoms are followed by gastrointestinal disorders, ocular abnormalities, and Gb3 accumulation, causing renal, cardiac, and neurological complications. The milder late-onset Fabry disease involves only a single organ system, typically the heart or the kidneys. Female Fabry patients have a mosaic expression for *GLA* as a result of X chromosome inactivation and they usually show less severe symptoms [32].

Increasing evidence suggests that cardiovascular morbidity is the main cause of death in Fabry patients, mainly due to increased risk of sudden cardiac death and heart failure [33]. The identification of serum biomarkers derived from collagen type I metabolism has been proposed to predict early fibrotic damage in Fabry patients to be followed by a prompt ERT procedure [34,35,36]. A second currently approved medication is based on chaperone therapy to correct the misfolded enzyme, but an increase in enzymatic activity and a decrease in Gb3/lyso-Gb3 accumulation does not occur in all patients. Currently, SRT and mRNA-based therapy are under evaluation [37].

### 2.3. Niemann–Pick Disease

Niemann–Pick disease (NPD) is an autosomal recessive inherited disorder due to hydrolase deficiency or impaired intracellular cholesterol trafficking. Mutations in acid sphingomyelinase (aSMase), encoded by *SMPD1*, are causative of the NPD type A and B forms, whereas NPD type C, a lysosomal storage disease distinct from sphingolipidoses, is a cholesterol trafficking defect due to mutations in *NPC1* or *NPC2* genes [38].

aSMase catalyzes the breakdown of sphingomyelin in ceramide and phosphocholine. The degree of severity of NPD type A and B depends on the aSMase residual activity owing to the type of *SMPD1* mutation [39]. When aSMase is mutated, its primary substrate accumulates in the monocytes and macrophages (foam cells) of the liver, spleen, lymph nodes, adrenal cortex, and bone marrow [40]. In children with NPD type A, foam cells infiltrate the brain, causing structural changes, gliosis, demyelination, and neuronal cell loss. Thus, NPD type A is the most severe form of NPD and death occurs within the second or third year of age. NPD type A has a high incidence in the Ashkenazi Jewish population, with a carrier frequency of 1 in 90, whereas NPD type B is a pan-ethnic disease characterized by a later onset and milder symptoms [40,41]. Currently, there is no efficacious treatment for NPD type A and B. Recombinant human aSMase selectively reduces sphingomyelin accumulation in NPD type B fibroblasts in vitro [42] and ERT is now under clinical trial [43].

NPD type C is due to mutations in *NPC1*, which encodes for a transmembrane protein of the lysosomal membrane, or *NPC2*, which encodes for an intracellular cholesterol transporter. Both deficiencies lead to intracellular accumulation of unesterified cholesterol and glycosphingolipids [44]. Its incidence is about 1 in 100,000 live births and can be divided into neonatal, late infantile, and juvenile [45]. Neonatal presentation is rare and characterized by progressive liver disease, which represents the most common cause of death among neonatal-onset NPD type C patients [46]. Late infantile and juvenile forms are the most common, characterized by the outbreak of neurological disorders; in contrast to the infantile form, there is no liver or spleen enlargement. NPD type C is usually treated with anti-hypercholesterolemic drugs, but this does not ease the symptoms [47,48]. 

### 2.4. Krabbe Disease

Also known as globoid cell leukodystrophy, Krabbe disease is an autosomal recessive disorder characterized by the deficiency of the acid hydrolase β-galactosylceramidase (*GALC*) encoded by the *GALC* gene. *GALC* catalyzes the removal of β-galactose from β-galactosylceramide (a major component of myelin) and other terminal β-galactose-containing sphingolipids, including the neurotoxic metabolite β-galactosylsphingosine (psychosine) [49].

By acting at different cellular levels, *GALC* deficiency causes psychosine accumulation paralleled by neuroinflammation, degeneration of oligodendroglia, and progressive demyelination [50]. Psychosine has been shown to inhibit protein kinase C signaling, activate the caspase cascade, disrupt the trans Golgi network and endosomal vesicles, and impair mitochondria and peroxisome function [51]. In addition, the detergent-like action of psychosine may disturb the membrane microdomain organization of lipid rafts, causing demyelination [51,52,53]. Moreover, deregulation of brain neovascularization occurs in Krabbe patients and in *twitcher* mice, an authentic model of the disease [54], whereas neuroinflammation leads to increased levels of long pentraxin 3, an innate immune response mediator that acts at the site of inflammation [55].

The early infantile form (onset at birth to 5 months of age) represents the most common and severe type of Krabbe disease. It is characterized by fast progression and the symptoms include regression of psychomotor development followed by seizures, loss of vision and hearing, and early death [56]. The late-infantile onset occurs between 13 and 36 months and is characterized by motor regression, ataxia, and progressive blindness [57]. Adult forms of Krabbe disease are rare; they display progressive spastic paraplegia and sometimes neuropathy [58].

ERT is not the most effective treatment because of its poor ability to pass the blood brain barrier and the immune response against the recombinant *GALC* protein [51]. Currently, the standard of care is HSCT, which significantly improves the lifespan of Krabbe patients when performed before the outbreak of symptoms [57].

### 2.5. Farber Lipogranulomatosis

Farber disease is a rare autosomal inherited metabolic disorder caused by inactivating mutations in the *ASAH1* gene that encodes for the lysosomal acid ceramidase. Acid ceramidase promotes the breakdown of ceramide in sphingosine and fatty acid, and its deficiency leads to the progressive accumulation of ceramide in bone, cartilage, immune system, central nervous system, lungs, and other organs [59]. Farber lipogranulomatosis has a wide range of age onset and clinical features, even though subcutaneous nodules, made of ceramide engorged macrophages, arthritis, and dysphonia are the three major signs of the disease [60]. As for other sphingolipidosis, the infantile form is the most severe, characterized by progressive neurologic regression and lung disorders. Milder forms present only modest or no alterations of the central nervous system [61]. Unfortunately, no effective therapies are currently available for this disease [59].

### 2.6. GM1 and GM2 Gangliosidoses

Gangliosides are glycosphingolipids that account for up to 10% of brain lipid content and were isolated from the human brain for the first time in 1939 by E. Klenk [62]. They are composed of sialic-acid-containing oligosaccharide chains linked via a β-glycosidic bond to ceramide, which is responsible for their insertion into cell membranes. Deficiencies in enzymes involved in their metabolism cause an accumulation of unmetabolized gangliosides in lysosomes, mainly in neurons where ectopic neurite outgrowth may occur [63].

#### 2.6.1. GM1 Gangliosidosis

β-Galactosidase is a lysosomal hydrolase that cleaves β-linked galactose residues from the non-reducing end of glycan moieties found in various glycoconjugates [62]. Deficiency in the β-galactosidase encoding gene *GBL1* leads to the accumulation of the GM1 ganglioside and its derivative GA1 mainly in lysosomes. Like all of the other lysosomal disorders, GM1 gangliosidosis is an inherited metabolic disease with an estimated incidence of 1 in 100,000–200,000 newborns [64].

The most severe form of the disease is the infantile type I GM1 gangliosidosis, characterized by hydrops fetalis developmental psychomotor regression and, as the child grows, hepatosplenomegaly and skeletal abnormalities. Type II GM1 gangliosidosis is named late infantile or juvenile, depending on the age at which the first symptoms arise: between 12 and 24 months for the late infantile form and 3–5 years for the juvenile form. Children quickly lose their ambulatory capacity and need a gastrostomy placement. In the juvenile form, ataxia and dysarthria follow the psychomotor decline [65]. The adult-onset type III GM1 gangliosidosis is characterized by milder and more varied symptoms, with a longer life expectancy [66].

Currently, no specific treatment exists for GM1 gangliosidosis; the therapy aims to relieve symptoms and is mostly palliative [67]. Recently, miglustat, a glucosylceramide synthase inhibitor, used for SRT in GD and NPD type C diseases [68,69], has also been proposed for the treatment of children affected by type II GM1 gangliosidosis [70]. 

#### 2.6.2. GM2 Gangliosidosis

The disease is due to the lysosomal accumulation of the GM2 ganglioside [71], which represents about 5% of all brain gangliosides [72]. The hydrolysis of GM2 to GM3 ganglioside is performed by β-hexosaminidase A (HEXA), a heterodimer whose α and β subunits are encoded by *HEXA* and *HEXB* genes, respectively, and requires the GM2 activator protein (GM2AP) as a cofactor [73]. In an ERT prospective, an enzymatically active recombinant protein homodimer HexM has been developed that is able to interact with the GM2AP–GM2 complex in vivo [73]. Currently, the use of HexM as ERT has not been transferred to the clinics and works are in progress to optimize an AAV vector for gene therapy [74,75] with reduced immune response reactions [76].

Three forms of GM2 gangliosidoses have been described: the AB variant, Tay–Sachs disease, and Sandhoff disease. They are characterized by neurological disorders that vary from hypotonia regression to cerebellar ataxia according to the age of onset [71].

##### AB Variant

The AB variant is the rarest form of GM2 gangliosidosis, with about 30 cases reported in the scientific literature. It is caused by inherited mutations of the *GM2A* gene that disrupt the activity of the GM2AP cofactor. The AB variant is characterized by severe cerebellar atrophy that causes dysphagia, muscle atrophy, psychotic episodes, and manic depression [72].

##### Tay–Sachs Disease

More than 130 mutations of the *HEXA* gene have been reported for Tay–Sachs disease, which has an incidence of 1 in 100,000 live births [77]. HEXA encodes for the α-subunit of the enzyme and the disease presents an ample heterogeneity of clinical symptoms based on hexosaminidase residual activity [69].

Tay–Sachs disease can be divided according to the age of onset. The infantile form represents the most aggressive form and is associated with very low hexosaminidase activity. Developmental delay arises around the sixth month of age and is followed by blindness, cognitive impairment, seizures, and paralysis, resulting in death before 5 years of age [78]. The juvenile form is characterized by ataxia, dysarthria, and developmental delay; the survival time is usually around 14 years [79]. The adult form is less severe and has 5–20% of hexosaminidase residual activity. With the progression of the disease, patients complain of leg weakness, ataxia, tremor, and psychiatric disorders [80]. Current treatments for Tay–Sachs patients involve SRT, bone marrow transplantation, hematopoietic or neural stem cell transplantation, and the use of anti-inflammatory drugs. However, most of the treatments have failed to relieve neurological symptoms owing to the difficulty in restoring hexosaminidase activity in the brain [77].

##### Sandhoff Disease

Sandhoff disease accounts for approximately 7% of GM2 gangliosidoses. In this type of GM2 gangliosidoses, *HEXB* variants prevent the correct catabolism of GM2 ganglioside with its lysosomal accumulation in the central nervous system and somatic cells [62]. 

As for other sphingolipidoses, Sandhoff disease has been classified into infantile, juvenile, and adult forms according to the severity of the disease and the age of onset. The cardinal clinical features of infantile Sandhoff disease are seizure, muscle weakness, developmental delay, and regression; death occurs before 3 years of age [81]. Late onset forms are less common and characterized by lower motor neuron disease and neurological degeneration [82,83]. Clinical manifestations, mainly in juvenile and adult Sandhoff patients, are heterogeneous and based on residual hexosaminidase activity. A case report of two siblings with compound heterozygous HEXB mutations further confirmed the clinical heterogeneity of Sandhoff disease [84]. As in Tay–Sachs disease, efficacious therapy for Sandhoff patients is still lacking owing to poor diffusion of the drugs into the nervous system [83].

### 2.7. Metachromatic Leukodystrophy

Metachromatic leukodystrophy (MLD) is an autosomal-recessive inherited sphingolipidoses caused by deficiency of the enzyme arylsulfatase-A encoded by the *ARSA* gene. The enzyme cleaves sulfatides in galactosylceramide and its deficiency leads to the formation of sulfatide-engulfed metachromatic granules in oligodendrocytes, microglia, Schwann cell, neurons, and macrophages, causing myelin degradation and inflammation [85]. Motor neurons derived from induced pluripotent MLD stem cells are characterized by lysosomal accumulation of sulfatides, mitochondrial fragmentation, and impaired autophagy, leading to premature cell death [86].

The worldwide incidence of MLD is around 1.5 in 100,000 live births, being much higher in Habbanite Jews (1:75) and Navajo Indians (1:2500) [85]. Different mutations in *ARSA* are associated with two groups with different residual arylsulfatase-A activity: the allele 459+1G>A is the most frequent mutation in Europe and belongs to group 0 with no residual activity, whereas the alleles 1277C>T and 536T>G represent the R group with minimal residual activity [87].

The disease can be also divided into four groups according to the age at onset: late infantile, early, and late juvenile, and adult forms. Late infantile and early juvenile MLD are the most frequent forms with severe and rapid progression; they arise during the second and fourth year of life, respectively, and the symptoms affect both the central and the peripheral nervous system [87]. Adult MLD is often misdiagnosed as early-onset dementia or schizophrenia because of its slow progression [85].

The most promising treatment is bone marrow transplantation or HSCT when performed before the onset of symptoms [85]. Moreover, HSCT leads to stabilization or reduced decline in motor and cognitive functions and the positive effects were particularly meaningful in the peripheral nervous system in patients with late-infantile MLD, refractory to other therapeutic interventions [88].

## 3. Sphingolipids in Zebrafish

**Figure 4:**
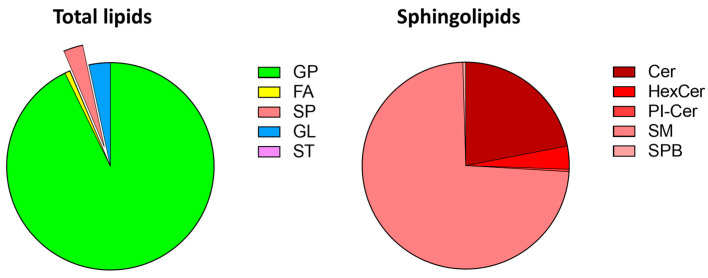
Lipid composition of zebrafish embryos. Untargeted lipidomic MS analysis of zebrafish embryos at 72 hpf identified 1377 lipid species. The relative amounts of the major classes of lipids are shown in the corresponding pie charts. Lipids were grouped into categories according to the LIPID MAPS consortium nomenclature [104]. Cer, ceramide; FA, fatty acyls; GL, glycerolipids; GP, glycerophospholipids; HexCer, hexosylceramides; PI-Cer, inositolphosphorylceramides; SM, sphingomyelins; SPB, sphingoid bases; SP, sphingolipids; ST, sterols.

Beginning with use as a vertebrate animal model during the 1980s [89], the teleost zebrafish has emerged as a useful platform for studies in diverse fields of research. The high grade of genome conservation between human and zebrafish (around 70%, and the percentage increases to 84% when focusing on genes associated with human diseases) [90], combined with precise genome editing and the ease of manipulation, enable to model several human diseases in zebrafish, such as cancer [91], neurodegenerative [92], cardiovascular [93], behavioral [94,95], and inherited [96,97] disorders, including sphingolipidoses. Indeed, lipidomic analysis has revealed the presence in zebrafish of all the principal lipid classes present in mammals (see [98,99] and Figure 4), supporting the possibility to model lipidic metabolism diseases in the fish with the advantage of using existing mammalian lipid databases for data processing [100]. In addition, zebrafish is useful to study lipidic changes after exposure to industrial pollutants [101], drugs [102], toxic compounds [103], or a high-cholesterol/high-fat diet [99]. Moreover, zebrafish larvae can be fed with fluorescent BODIPY-lipids to serve as metabolic tracers when incorporated in vivo into more complex lipid products [99].

### 3.1. Zebrafish Lipidomics

The dynamic composition of lipids in the body and yolk sac of zebrafish embryos was investigated during the first 5 days of development via liquid chromatography/mass spectrometry (LC/MS), demonstrating significant differences between the two embryonic compartments [98]. The results have shown that cholesterol, phosphatidylcholine, and triglycerides are the most abundant lipids in zebrafish embryos. Of note, the yolk not only represents simple storage of lipids to provide energy for the growing embryo, but also an active organ where lipids are remodeled before reaching the embryo body.

Desorption electrospray ionization MS imaging followed by nanoelectrospray MS and tandem MS (MS/MS) were used to detect phosphatidylglycerols, phosphatidylcholines, phosphatidylinositols, free fatty acids, triacylglycerols, ubiquinone, squalene, and other lipids during zebrafish embryonic development from 0 to 96 h post fertilization (hpf) [105]. In addition, high-spatial-resolution matrix-assisted laser desorption/ionization (MALDI) MS imaging was applied to map and visualize the 3D spatial distribution of phosphatidylcholine, phosphatidylethanolamines, and phosphatidylinositol molecular species in zebrafish embryos at the one-cell stage, whereas high-spatial-resolution 2D MALDI MS imaging was used to analyze zebrafish embryos at the 1- to 16-cell stages [106]. These studies have allowed to investigate the composition and distribution of lipids in zebrafish, with insights about lipidic dynamics during embryonic development. 

Given the growing interest in the study of the zebrafish lipidome, attempts have been performed to improve the quality of lipid analysis. For instance, conventional one-dimensional LC (1D-LC) was compared to comprehensive two-dimensional LC (2D-LC) coupled to a high-resolution time-of-flight mass spectrometer for a full-scale lipid characterization of lipid extracts from zebrafish embryos. The results demonstrate that 2D-LC is 2.5 times more efficient than 1D-LC, allowing the annotation of more than 1700 lipid species [107].

Recently, a direct infusion MS/MS approach using multiple reaction monitoring was applied to precisely quantify membrane lipid composition both in the yolk and in the zebrafish embryo body during the gastrula stage [108]. Around 700 membrane lipids were annotated, divided into two main lipid classes: sphingolipids and phospholipids, with the latter including phosphatidylcholine, phosphatidylinositol, phosphatidylserine, and phosphatidylethanolamine. The composition of the embryo body and yolk was quite similar, with phosphatidylcholine representing the most abundant species. However, major differences were found in the content of phosphatidylserine, dihydrosphingolipids, and sphingomyelin with short-chain fatty acids (significantly higher in the embryo body than in the yolk). Notably, the fine tuning of the sphingolipid synthesis appears to be related to the *wnt* pathway and is fundamental for proper orientation during cell division.

Lipidomic analysis can also be applied to specific organs from adult zebrafish. For instance, livers from 6-month-old animals were analyzed with different MS techniques, identifying 712 unique lipid species from four categories (fatty acyls, glycerolipids, glycerophospholipids, and sphingolipids) [109]. Moreover, adult zebrafish brains have been analyzed for changes in the lipid profile after exposure to different xenobiotics [102,110].

The central hub of the sphingolipid pathways ceramide and its derivatives play a pivotal role in different biological processes [3]. The ceramide profiles of adult zebrafish brain, 7-day-old zebrafish larvae, and human cells were compared using a parallel reaction monitoring approach in which a targeted quantification method was associated with high-resolution hybrid MS [111]. The results highlighted a significant overlapping in ceramide distribution, even though a scarcity of sphingadiene-containing ceramides was observed in zebrafish specimens, despite their biological importance in mammals. These results raised the hypothesis about possible alternative unexplored lipidic pathways in zebrafish that might pave the way for novel discoveries in human sphingolipid disorders. Targeted sphingolipidomics performed at various stages of embryonic development and in adult animals under different physiological and pathological conditions are required for a better understanding of sphingolipid metabolism and function in zebrafish.

### 3.2. Sphingolipid Metabolizing Enzymes in Zebrafish

Zebrafish and human genomes share a high homology [90], thus several lipid-metabolizing enzymes involved in human diseases have a zebrafish counterpart (Figure 2).

Ceramide synthases (*CERS*), the enzymes responsible for ceramide production, play a central role in the sphingolipid metabolism. Highly conserved through evolution, the *CERS* gene family encompasses six isoforms (*CERS1–6*) with diverse spatial/temporal expression in mammals. All *CERS*s except for *CERS*1 show an *N*-terminal homeobox-like domain whose functions remain elusive [112]. In zebrafish, nine genes encoding for the six *cers* subtypes have been identified with a sequence homology with human and mouse counterparts ranging from 46% to 79% identity. Owing to the genome duplication typical of zebrafish, *cers2*, *cers3*, and *cers4* are present as double copy genes (*a* and *b*), while *cers1*, *cers5*, and *cers6* are present as single copy genes. As in mammals, all zebrafish orthologs display the Hox domain, except for the Cers1 protein.

The tissue-/stage-dependent expression of the *cers* genes has been analyzed during zebrafish embryo development by whole mount in situ hybridization (WISH) [113]. The results suggest that these enzymes are involved in diverse biological processes and that the production of ceramides may dynamically vary in different tissues. For instance, only *cers2a* and *cers3b* are expressed in the embryonic zebrafish pronephros, congruent with the high expression of *Cers2* in murine kidney, while all *cers* are expressed in the nervous tissue, possibly pointing to the requirement for various ceramide species in the developing brain. Notably, the expression of *cers* can be modulated in zebrafish embryo when a perturbation in the lipidic composition occurs. Indeed, zebrafish embryo mutants for the sphingosine kinase gene *sphk2*, in which a potentially dangerous accumulation of the metabolite sphingosine occurs, upregulate the expression of *cers2b* to activate the sphingolipid salvage pathway and turn the excess of sphingosine in ceramide [114]. In this frame, the ortholog of the human peroxisome proliferator-activated receptor γ-responsive transmembrane gene *FAM57B*, involved in the regulation of ceramide metabolism, was found to maintain the homeostasis of sphingolipids and glycerol lipids during brain development in zebrafish [115]. Indeed, untargeted lipidomic analysis performed in the brain tissue of 7-day-old *fam57b* null and heterozygous zebrafish lines has revealed remarkable differences in the lipid profile with consequences on membrane composition and permeability when compared with wild type animals.

As described above, ceramide catabolism is catalyzed by the lysosomal acid ceramidase encoded by the *ASAH1* gene. In silico analysis has revealed the presence of two *ASAH1* co-orthologs in zebrafish (*asah1a* and *asah1b*) [116]. Genome editing techniques have revealed the importance of lysosomal acid ceramidase to maintain the physiological levels of ceramide in zebrafish. Indeed, *asah1a* and *asah1b* enzymes are both able to hydrolyze ceramide and the presence of either *asah1a* or *asah1b* prevents substrate accumulation, with ceramide being increased only in double *asah1a^−/−^*/*asah1b^−/−^* mutants [117].

Ceramide represents the substrate for the generation of more complex sphingolipids, such as sphingomyelin, a central component of myelin. The enzymes responsible for the production of sphingomyelin from ceramide are named sphingomyelin synthases (*SMSs*). As reported in the ZFIN database [118], two duplicated genes are predicted for the two human *SMS* genes in zebrafish (*sgms1a*, *sgms1b*, *sgms2a*, and *sgms2b*). Moreover, a gene ortholog for the human enzymatically inactive *SMS*-related protein (*SMSr*) has been reported in zebrafish, named *zgc:175139* [119]. *SMSr* represents a key regulator of ceramide homeostasis that may operate as a sensor rather than a converter of ceramides in the ER [120]. However, its role in zebrafish remains unexplored.

SMases, also named sphingomyelin phosphodiesterases, catalyze the production of ceramide and phosphocholine from sphingomyelin, representing one of the three ceramide production pathways together with the de novo synthesis and the salvage pathways [121]. In zebrafish, *smpd1* has been identified as a single ortholog of the aSMase-encoding gene *SMPD1*, which shares 59% identity with the human counterpart. A mutant line of *smpd1* was created in zebrafish via the CRISPR/Cas9 technique; the SMase enzymatic activity was abolished by 93% at 5 days post fertilization (dpf) with a consequent increase in various sphingolipid metabolites [122].

*SMPD2* encodes for the membrane-bound Mg^2+^-dependent neutral SMase1. Its *smpd2* ortholog has been cloned in zebrafish and it has been shown to mediate ceramide production and activation of apoptosis following heat stress in zebrafish embryonic cells [123]. Of note, neutral SMase1 is activated by phosphorylation at Ser-270 downstream of the c-Jun N-terminal kinase pathway in both zebrafish and human cells [124], and thalidomide exerts an antiangiogenic effect on zebrafish embryos due to the upregulation of neutral SMase activity and the consequent production of ceramides [125]. In addition, a mitochondrial neutral SMase (mtSMase) has been characterized in zebrafish. mtSMase was purified from zebrafish cells and tested for its enzymatic activity, showing an optimum working pH of 7.5 and sphingomyelin as the main substrate. Cell fractionation and immunofluorescence analysis demonstrated the mitochondrial localization of this novel SMase. Another neutral SMase has been identified in zebrafish that represents the ortholog of the human gene *SMPD3* with a conserved identity of 55% [126].

The sphingoid base sphingosine is an important component of sphingolipid metabolism. Its biologically active metabolite sphingosine-1-phosphate (S1P) is involved in a variety of physiological and pathological processes by binding specific G-coupled receptors (S1PRs) [127]. The study of sphingosine and related metabolites in zebrafish can provide novel information about the human counterparts, favoring a better understanding of the biological mechanisms involved in sphingolipidoses and other pathologies. Two S1P phosphatase (*spp1* and *spp2*), two sphingosine kinase (*sphk1* and *sphk2*), and one sphingosine lyase (*spl*) encoding genes have been identified in zebrafish, together with seven conserved *s1pr* orthologs corresponding to the five human *S1PR*s (*s1pr3* and *s1pr5* being duplicated in zebrafish). *s1pr1* is highly expressed in the brain, while *s1pr4* is expressed mainly in the kidney, which represents the zebrafish hematopoiesis site, thus reflecting the mammalian *S1PR* expression in lymphoid and hematopoietic tissues [128]. Knockdown (KD) and knockout (KO) approaches have shown that the S1P/S1PR pathway plays a pivotal role in vascular and cardiac development in the zebrafish embryo [129,130].

A single ortholog of the human *GLA* gene is present in zebrafish (*a-gal*) with significant similarities between human and zebrafish proteins (>70%). [131]. Enzymatic and immunohistochemical analyses have shown that the zebrafish protein retains significant α-galactosidase activity and a distribution in zebrafish kidney like in humans, suggesting that it may retain the same biological functions. Finally, the zebrafish gene *arsa* is reported in the ZFIN database [118] as an ortholog of the human gene *ARSA* with a predicted arylsulfatase activity; however, its role in zebrafish remains to be investigated.

## 4. Zebrafish as an Animal Model for Sphingolipidoses: The “Zebra-Sphinx” Platform

In the last decades, the use of zebrafish to study gene function has increased exponentially thanks to the multiple advantages offered by this model, such as a high number of offspring generated, embryo transparency, and quick genetic manipulation. The necessity to target distinct genes to study their function led to the development of different techniques to block gene function either in a transient manner or permanently. One of the most used commonly techniques involves the injection of antisense oligonucleotides complementary to specific genetic loci, named morpholinos (MOs), which temporarily KD protein production [132]. There are two different strategies by which MOs can interfere with protein expression. The first strategy is based on the block of the translation of the targeted gene (ATG-MO). The second one is aimed at interfering with the splicing process that occurs during mRNA maturation (splicing-MO) [96]. 

Genome editing techniques have been extensively applied in the zebrafish field. To date, the most used techniques are the Zinc-Finger Nuclease, the Transcription Activator Like Effector Nuclease (TALEN) [133,134], and a more recent approach based on the CRISPR/Cas9 system [135,136,137]. Briefly, these systems use different approaches to drive proteins with nuclease activity to a specific DNA sequence. Once bound to the locus, the nucleases cut the double-stranded DNA, forcing the cell to activate double-strand break repair mechanisms. The repair process mediated by the non-homologous end-joining repair system can introduce deletions or insertions (indels) into the break-point region, which can lead to alterations of the reading frame and hence to an altered protein sequence and loss of gene function. Using these procedures, several models of inherited human diseases have been generated in zebrafish. In line with this review, we will discuss the phenotypes of the main models of sphingolipidoses in zebrafish (Table 2).

### 4.1. Gaucher Disease

Injection of a splicing-MOs directed against *gba1*, the zebrafish ortholog of the human *GBA1* gene, caused the appearance of specific alterations in 5 dpf embryos, including curvature of the trunk, defects of primary bone ossification with a decrease in *col10a1* and *runx2b* gene expression associated with a dysfunction of the osteoblast population, and severe erythropenia and thrombocytopenia caused by early hematopoietic defects. Microarray analysis demonstrated alterations in the expression of genes involved in different biological processes, including mitochondrial activity and intracellular vesicle trafficking. These defects were paralleled by increased oxidative stress and reduced signaling of the Wnt/β-catenin pathway [138]. In a different study, *gba1* KD led to an increased number of vacuolated macrophages, characterised by migratory defects and enlarged lysosomes, pointing to an impairment in the macrophage function due to the low levels of acid β-glucocerebrosidase activity [148].

The first KO model for GD was derived from a forward genetic screening that led to the identification of a *gba1^sa1621/sa1621^* mutant zebrafish line. The characterization of this mutant revealed a decrease in the body length and curvature of the spine at 7 dpf [138]. Like *gba1* morphants, *gba1^sa1621/sa1621^* mutants show a reduction in *col10a1* and *runx2b* expression related to osteoblast function, as well as erythropenia. In addition, splenomegaly and hepatomegaly can be observed in 3-month-old mutants.

To date, different zebrafish KO models of GD have been generated taking advantage of genome editing techniques. The first engineered *gba1* null mutant was obtained by TALEN approaches [139]. Mutants did not show any significant defect during the early stages of development, with the first alterations in the swimming behaviour occurring at 8 weeks of age and curvature of the spine at 12 weeks. Histopathological analysis revealed the presence of Gaucher-like cells in the brain, liver, thymus, and pancreas of adult KO animals. Furthermore, dopaminergic neuron degeneration, the presence of cytoplasmic inclusions resembling Lewy bodies, an increased number of autophagosomes, and microglia activation were observed in KO brains. MS demonstrated the accumulation of sphingolipid metabolites in *gba1*^−/−^ larvae at 5 dpf. They included hexosylsphingosine, glucosylceramide, lactosylceramide, and galactosylceramide, and their levels were further increased in juvenile brains.

A second zebrafish model of GD was generated by the CRISPR/Cas9 technique [140]. As observed for the *gba1* null zebrafish generated by TALEN, adult *gba1* KO mutants showed a curved back, and swimming and feeding impairment starting from 10 weeks of age. In addition, adults were characterized by the presence of Gaucher-like cells in the liver, spleen, and pancreas, together with an increase in glucosylsphingosine and glucosylceramide levels in the brain and liver. However, in this study, *gba1* KO larvae appeared to accumulate solely glucosylsphingosine at 5 dpf, and no altered levels of other glycosphingolipids were observed at this stage. Expression analysis of specific mRNAs in the brain of adult *gba1*^−/−^ zebrafish mutants [117] revealed the upregulation of macrophage (*gpnmb*, *chia.6*), microglia (*apoeb*), and complement system (*c1qa*, *c3.1*, *c5*, *c5aR1*) markers, as well as the upregulation of proinflammatory cytokines (*il1-b*, *tnf-a2*), whereas downregulation of the dopaminergic neuron marker (*th1*) and of the myelin-related gene (*mbp*) were observed. Autophagy was also increased in these brains.

More recently, a further KO model was generated in zebrafish using TALEN [151]. Animals showed a reduction in dopaminergic and noradrenergic neurons at 3 months of age, confirming the importance of *gba1* function for neuronal survival.

Cytosol-facing GBA2 metabolizes cytosolic glucosylceramide. Genetic ablation of the *Gba2* gene exerts beneficial effects in murine models of GD and NPD type C [152,153]. In order to investigate the potential role of GBA2 in compensatory glucosylceramide metabolism during inadequate GBA1 activity, double *gba1* and *gba2* null animals were generated by CRISPR/Cas9 in zebrafish [140]. Lipid analysis performed on double mutants at 5 dpf showed increased glucosylceramide levels when compared with single *gba1*^−/−^ larvae, but similar to those detected in single *gba2*^−/−^ animals. Moreover, glucosylcholesterol was significantly decreased in the double KO animals and in single *gba2*^−/−^ mutants. In addition, a significant accumulation of glucosylsphingosine occurs in double *gba1*/*gba2* null animals when compared with controls. Notably, in keeping with an SRT approach for the treatment of GD, the administration of the potent specific glucosylceramide synthase inhibitor eliglustat elicited a significant decrease in hexoxylceramide and in the derived lipids glucosylsphingosine and hexoxylcholesterol in *gba1*^−/−^ larvae. Together, these data indicate that zebrafish larvae offer an attractive model to study glucosidase actions on glycosphingolipid metabolism in vivo.

To study the role of excessive glucosylsphingosine formation during acid β-glucocerebrosidase deficiency, KO zebrafish lines were generated for the two *ASAH1* orthologs *asah1a* and *asah2b* [117]. Of note, double *asah1a*/*asah1b* null larvae, but not single *asah1a* or *asah1b* mutants, accumulate the primary substrate ceramide. Nevertheless, only *asah1b* appears to be involved in the formation of glucosylsphingosine in a *gba1*-deficient background. Accumulation of glucosylsphingosine in *gba1*^−/−^*/asah1b^−/−^* zebrafish did not prevent the formation of Gaucher-like cells, glucosylceramide accumulation, or neuroinflammation. However, these double mutants show an ameliorated course of disease reflected by a delay in the appearance of locomotor abnormalities and curvature of the back, reduced loss of dopaminergic neurons, and increased lifespan, suggesting that the accumulation of glucosylsphingosine may play a role in the pathogenesis of GD.

A similar approach was used to investigate the impact of acid SMase activity on a glucocerebrosidase-deficient background by generating double *gba1*^−/−^*/smpd1*^−/−^ zebrafish mutants [122]. Unexpectedly, double *gba1*^−/−^*/smpd1*^−/−^ mutants showed a markedly prolonged survival, rescue of neuronal and mitochondrial damages, and normalization of the motor phenotype when compared with *gba1* KO animals. This occurred in the presence of an additive increase in the levels of various sphingolipid metabolites.

Both *GSC1* and *SMPD1* variants represent inherited risk factors for Parkinson’s disease [154]. In keeping with the data obtained in double KO animals, human cells with combined glucocerebrosidase and aSMase deficiency showed an unpredicted reduction in intracellular α-synuclein levels. Together, these observations indicate that a better understanding of the crosstalk among sphingolipid metabolizing enzymes is required to dissect the pathogenesis of sphingolipid-related pathologies and for the development of efficacious therapeutic approaches.

### 4.2. Fabry Disease

A model of Fabry disease was obtained in zebrafish by the generation of a *α-gal* KO fish line using the CRISPR/Cas9 technique [131]. This led to the decrease in α-gal protein expression in the kidney associated with a marked reduction in α-galactosidase activity. Even though KO mutants did not show significant differences in body size when compared with wild type animals, they were characterized by an increased mortality during the early embryonic stages. A more in-depth analysis unveiled an increase in creatinine levels and the leak of high molecular weight proteins, suggesting that an impairment of glomerular filtration may occur in these mutants. Accordingly, microscopic analysis of the kidney revealed an increased glomerular size, dilated capillary loop, and thinner Bowman’s space. In contrast with the results obtained in other animal models of Fabry disease that do not show renal abnormalities [155], these data are in keeping with the nephropathy that occurs in Fabry patients [156]. Notably, the absence of a Gb3 synthase encoding gene ortholog in zebrafish provides the unique opportunity to identify pathogenic processes that may work in concert with Gb3 accumulation in Fabry disease [131]. 

### 4.3. Niemann–Pick Disease

A KO model of NPD type A was generated in zebrafish using the CRISPR/Cas9 system [122]. *Smpd1* KO animals showed a 93% reduction in the enzymatic activity at 5 dpf, with a consequent increase in sphingolipid metabolites, including sphingomyelin, ceramide, lactosylceramide, and sphinganine. However, despite the absence of enzyme activity and the significant increase in key glycolipid substrates, no obvious phenotype was observed in embryo and adult KO animals.

At variance with the paucity of zebrafish models for the aSMase-deficient forms of NPD, various attempts have been made to model the type C form of NPD, a lysosomal storage disease distinct from sphingolipidoses that depends on cholesterol trafficking defects due to mutations in *NPC1* or *NPC2* genes. For instance, injection of specific MOs for *npc1*, the orthologous of *NPC1*, induces an accumulation of unesterified cholesterol at early embryonic stages [141]. Morphological evaluation of the zebrafish KD morphants injected at one cell stage or in the yolk syncytial layer revealed a disorganization of the actin cytoskeleton and a delay in the development during epiboly, unveiling a role for *npc1* in cell movement at this embryonic stage. Interestingly, a lower dose of MO was associated with a milder phenotype characterized by neuronal death, like in the human pathology. KD of *npc1* in zebrafish has also been associated with thrombocytopenia, as observed in some NPD patients [157]. 

Various KO models for NPD type C have been developed using the CRISPR/Cas9 technology to inactivate the *npc1* [142,143] or the *npc2* [144] gene. KO of *npc1* caused premature death of half of the animals during the embryonic and juvenile stages, with a significant reduction in body length, together with hepatomegaly, splenomegaly, neurological defects, and ataxia—features that resemble those observed in patients and other animal models of NPD type C. Moreover, analysis of hepatocytes unveils a massive accumulation of cholesterol and changes in the levels of different types of lipids, including ceramide, diacylglycerol, and lysophosphatidic acid [144,145]. At variance with *npc1* null animals, *npc2* KO fishes were able to reach adulthood, even though they showed a reduction in body size and impairment in the locomotor system starting from 2 months and 4 months of age, respectively [144]. Histopathological analysis of *npc2* KO adults revealed the presence of foam cells in liver and kidney, defects in axonal myelination, and alterations of cerebellar Purkinje cells. Notably, significant alterations have also been observed in *npc2* null zebrafish at early stages of development [145]. They include the accumulation of unesterified cholesterol, upregulation of markers of inflammation and activated microglia, mitochondrial dysfunction, defects in the myelination process, and an anxiety-like behaviour. Like what was observed in *npc1* maternal mutants, *npc2* KO derived from homozygote females show an aberrant phenotype already at 30 hpf, such as a curved tail, absence or abnormalities of the otoliths, defects in the brain structures, and lack of circulating cells—defects that may arise from an impairment of the Notch3 signaling pathway [145]. 

As for NPD type C, zebrafish has been used as a platform to model lysosomal storage diseases other than sphingolipidoses, including mucolipidosis type II (MLII) and mucopolysaccharidosis type II (MPSII), providing novel information about the pathogenesis of these disorders. 

Briefly, MLII is due to the mutation in the *GNPTAB* gene, encoding for the catalytic subunit of *N*-acetylglucosamine-1-phosphotransferase that catalyses the first step of the formation of mannose 6-phosphate (M6P)-tagged lysosomal soluble hydrolases. As a consequence of *GNPTAB* mutation, the lack of the M6P tag causes the missorting and secretion of such hydrolases, with lysosomal accumulation of their substrates. A first zebrafish model of MLII was obtained by MO injection. Morphant embryos showed craniofacial defects, impaired motility, and abnormal otolith and pectoral fin development. This model allowed to undercover alterations in the spatial-temporal expression of type II collagen and Sox9 [158]. Stable mutant lines for the *gnptab* gene were obtained by TALEN and site-directed mutagenesis technologies. Zebrafish mutants showed a craniofacial phenotype and elevated levels of cathepsin K activity associated with abnormal cartilage development and heart and valve malformations [159,160].

MPSII is caused by mutation in the *IDS* gene, encoding for the lysosomal enzyme iduronate 2-sulfatase, leading to the toxic accumulation of glycosaminoglycans into lysosomes (mainly dermatan and heparan sulphates) and multi-organ damage. An MO approach in zebrafish targeting *ids*, the single ortholog for human *IDS*, caused early defects in embryonic development. In particular, the abnormal migration and differentiation of neural crest cells into chondroblasts were responsible for craniofacial cartilage defects, while sonic hedgehog pathway disruption led to congenital heart defects [161,162]. In addition, KO of *ids* in zebrafish has provided novel information about the role of early deregulation of the fibroblast growth factor signaling pathway in the occurrence of irreversible skeletal defects before glycosaminoglycans’ accumulation [163]. With a different approach, human-mutated *IDS* mRNAs have been injected into zebrafish embryos for a rapid preliminary study about novel *IDS* point mutations associated with MPSII [164].

### 4.4. Krabbe Disease

Two *GALC* co-orthologs have been identified in zebrafish (*GALCa* and *GALCb*) that share a high identity with their human counterpart [148]. Further analysis confirmed that both isoforms are endowed with enzymatic activity and are localised in the lysosome. Moreover, WISH analysis revealed their co-expression in the central nervous system during embryonic development. Injection of single *GALCa* or *GALCb* specific MOs in zebrafish resulted in the partial reduction in enzymatic β-galactosylceramidase activity, which was completely abolished by the simultaneous injection of both MOs. Nevertheless, no evident morphological alterations were observed in both single- and double-injected morphants during embryonic development. Notably, no alterations in psychosine levels were detected in double *GALCa/GALCb* morphants, suggesting that the transient abrogation of *GALC* activity is not sufficient to accumulate this metabolite [146].

Relevant to this point, myelination in zebrafish starts in the hindbrain at day 4 of development and is not completed at day 10 [165], making the study of the effect of β-galactosylceramidase deficiency on myelination in zebrafish morphants unfeasible. However, analysis of the expression pattern of a set of neuronal marker genes unveiled a significant reduction and partial disorganization in *neurod1* expression and neuronal death in double *GALCa/GALCb* morphants, in keeping with the neurodegenerative features of Krabbe disease. These data suggest that *GALC* loss-of-function may have pathological consequences independent of psychosine accumulation, thus providing new insights into the pathogenesis of Krabbe disease. This possibility is supported by the observation that psychosine levels do not correlate with nervous system regions exhibiting demyelination and axonopathy in *twi-5J* mice harboring a spontaneous missense *GALC* mutation [166]. Thus, double *GALCa*/*GALCb* zebrafish morphants may represent an interesting option for addressing previously unrecognized psychosine-independent key aspects of the pathogenesis of Krabbe disease.

### 4.5. Farber Lipogranulomatosis

Transient downregulation of *asah1b* using an ATG-MO approach led to a 74% decrease in acid-ceramidase activity in zebrafish embryos [147]. Embryo morphants develop macroscopic phenotypic alterations by 48 hpf. Further analysis has disclosed increased neuronal apoptosis localised only in the spinal cord, leading to a reduction in the number of motor neuron branches. This defect does not affect peripheral projections, indicating a specific susceptibility of motor neurons to the reduced levels of lysosomal acid-ceramidase [147].

A more recent zebrafish model of Farber disease was generated using the CRISPR/Cas9 technique [111]. Three different mutant lines were generated: single KOs for each of the two co-orthologs (*asah1a* or *asah1b*) and a double *asah1a*/*asah1b* KO. At variance with the MO model, the abrogation of only one gene (*asah1a* or *asah1b*) did not lead to the appearance of an evident phenotype until adulthood, whereas double KO animals display a progressive reduction in body size when compared with wild type and single KO siblings. These differences became more evident 3 months after birth and double KO animals died within 4 months [111]. Accordingly, sphingolipid analysis performed on the brain of 3.5-month-old fishes revealed a significant accumulation of ceramide only in double KO animals. These results indicate that the activity of a single *asah1* ortholog is sufficient to maintain physiological levels of ceramide and to guarantee a normal phenotype in zebrafish. At variance, reminiscent of the joint deformations observed in Farber patients [61], the complete abrogation of acid-ceramidase activity impairs the normal growth of the skeletal system in zebrafish and induces a premature death, probably due to heart failure or seizure related to progressive ceramide accumulation.

### 4.6. GM2 Gangliosidoses

#### 4.6.1. Tay–Sachs Disease

During a multi-gene analysis to understand the role of macrophages in tuberculosis progression, a zebrafish model for Tay–Sachs disease was generated by injecting an MO targeting *hexa*, the ortholog of human *HEXA*. This model was characterized by augmented macrophages that show migratory defects and enlarged lysosomes [148].

#### 4.6.2. Sandhoff Disease

Analysis of different MOs in a wide range screening for angiogenesis inhibitors in zebrafish revealed that downregulation of *hexb*, the *HEXB* ortholog, induces defects in the vascular system at 48–56 hpf, as shown by FITC-dextran microangiography and by WISH analysis of the expression of the endothelial cadherin-5 encoding gene *cdh5* in the intersegmental vessels [149].

More recently, a KO model of Sandhoff disease was generated using a CRISPR/Cas9 approach targeting *hexb* [150]. The enzymatic activity of *hexa*^+/+^/*hexb*^−/−^ animals was reduced by 99% compared with controls, indicating that the *hexa* does not contribute significantly to the total β-hexosaminidase activity in zebrafish. Despite the lack of enzymatic activity, *hexb* null adult fishes are viable and show a normal morphological phenotype. However, mutant fishes showed an accumulation of different oligosaccharides in the brain and various internal organs. A more in-depth analysis performed on *hexb* KO embryos at 5 dpf evidenced abnormality in the lysosome morphology of the microglia and radial glia, probably associated with defects in the lysosome fusing process. A behavioural analysis of 4.5 and 6 dpf embryos showed a reduced locomotor activity of *hexb* KO animals compared with controls, an alteration that resembles the impaired locomotor function observed in Sandhoff patients [167]. Interestingly, the manifestation of this locomotor alteration is simultaneous with the appearance of lysosomal abnormalities in the radial glia, suggesting a correlation between glial function and locomotor activity. Moreover, *hexb* KO animals exhibit a slight increase in neuronal loss at 5 dpf that partially mimics the neurodegeneration observed in humans.

### 4.7. Metachromatic Leukodystrophy

The only zebrafish model established so far for MLD was obtained by injection of a splicing-MO specific for the *arsa* gene [148]. An initial characterization of this KD model showed an increased number of vacuolated macrophages presenting enlarged lysosomes compared with control embryos. Moreover, as observed in *gba1* and *hexa* null animals, abnormal macrophages showed an impairment of movement associated with migratory defects. These results indicate that diverse lysosomal storage disorders may impair macrophage function with an impact on their anti-microbial function [148].

## 5. Concluding Remarks

**Figure 5:**
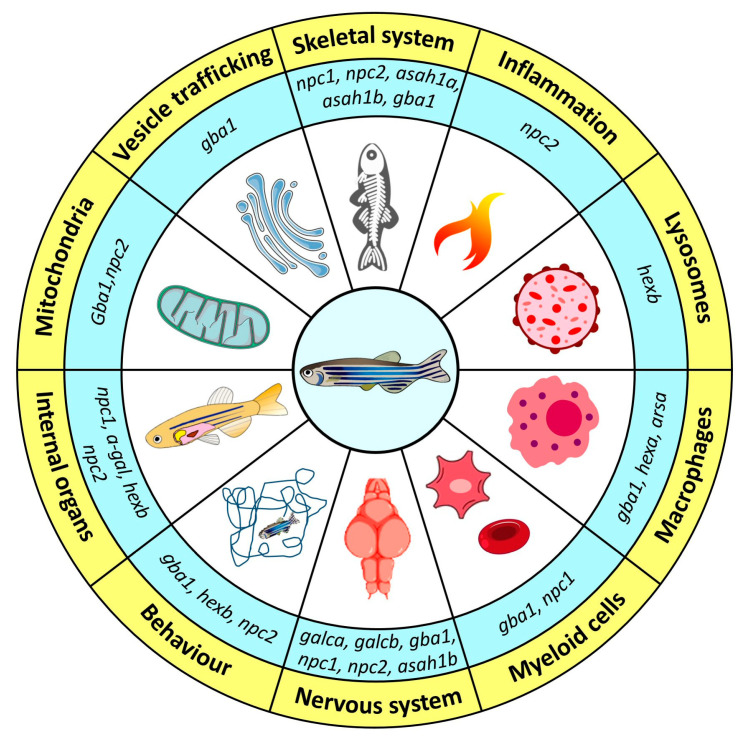
Main phenotypic alterations in zebrafish models of sphingolipidoses. Schematic summary of the major biological systems affected in zebrafish embryos or adults following the knockdown or knockout of the indicated genes encoding for different sphingolipid metabolizing enzymes. See text for details.

In this review, we have highlighted the use of zebrafish to develop new animal models of sphingolipidoses. Starting from a gene knockdown approach via MO injection at the one–two cell stage of embryonic development, the more recent use of the TALEN and CRISPR/Cas9 gene editing techniques has allowed to knock out enzymes involved in sphingolipid metabolism whose deficiency is responsible for various human hereditary sphingolipid disorders. Notably, many of these models recapitulate, at least in part, the phenotypic defects observed in patients (Figure 5). In addition, lipidomic analysis has allowed the study of the impact of enzymatic deficiencies on the sphingolipid metabolism in zebrafish, providing useful insights into the pathogenesis of these diseases. It must be pointed out that these studies can be performed not only in adult animals, but also in zebrafish embryos, thus providing invaluable information about the early biochemical alterations that may occur in patients before birth. 

At present, different therapeutic approaches, including HSCT, ERT, SRT, pharmacological chaperones, and in vivo and ex vivo gene therapy, are envisaged for patients affected by sphingolipidoses. However, given the complexities resulting from the alterations of sphingolipid metabolism in different systemic organs and the early appearance of serious pathological alterations in the infantile forms of various sphingolipidoses, more efficacious therapeutic strategies are required to improve patient outcomes.

As described in numerous reviews [96,97], the zebrafish system includes several advantages that make this organism a powerful platform for the study of the pathogenesis of human hereditary diseases and for the development of novel drug-based therapeutic strategies. Indeed, as also pointed out in this review, most of the pathogenic processes of genetic diseases are conserved between humans and zebrafish, with high similarity among possible drug targets. Compared with cell-based and biochemical screening of putative drug candidates, zebrafish models offer the great advantage of providing a whole organism response to the delivery of drug candidates, thus also allowing the evaluation of side effects such as teratogenicity, toxicity, and metabolic alterations, as well as the study of drug pharmacokinetics and pharmacodynamics [168]. In addition, the zebrafish embryo offers multiple advantages that make this model attractive for a cost-effective drug screening, including external fertilisation, high fecundity, and ease of use; furthermore, embryo transparency enables imaging at cellular resolution and internal organ visualization. Given these features, zebrafish has been used as a tool for high-throughput screening of different drug candidates relevant to a broad range of human diseases [169]. Many of these molecules have reached the clinical trial phase, confirming the possibility of using zebrafish as a platform for the development of new potential therapeutic strategies.

In this frame, even though some of the approaches envisioned for the therapy of sphingolipidoses cannot be modelled in zebrafish (like HSCT and gene therapy), various studies indicate the possibility to assess, in KO zebrafish mutants, the therapeutic potential of novel drugs to be used in an SRT approach. In addition, double KO zebrafish mutants harbouring the pathogenic mutation together with the genetic deficit of an upstream enzyme involved in the synthesis of the accumulating substrate may provide useful information about the possible efficacy of drug-driven SRT strategies. Moreover, zebrafish models of sphingolipidoses might be useful for the screening of pharmacological chaperones in zebrafish lines obtained by CRISPR/Cas9-based point mutation gene editing that harbour the identical pathogenic mutation detected in human patients.

Clearly, given the obvious differences with humans, zebrafish models may not fully reflect the pathophysiology of the human disease. In addition, the duplication of the zebrafish genome may result in the presence of two co-orthologs of the human pathogenic gene. This may require the understanding of the biological role of both proteins encoded by the two orthologs during zebrafish development and in adults to evaluate how and to what extent the single or double KO mutants may mimic the human disease. Despite these and other drawbacks, the “zebra-sphinx” system represents an innovative and informative tool to gain insights into the biology of sphingolipid metabolism for a better comprehension of the pathological processes contributing to sphingolipid disorders, thus enabling the development of novel potential therapies and their translation to patients.

## Figures and Tables

**Table 1:** Human sphingolipidoses.

Disease	OMIM	Affected Gene	Deficient Protein	Main Accumulated Metabolite
Gaucher	#230800 (type I) #230900 (type II) #231000 (type III)	*GBA1*	Acid β-glucocerebrosidase	Glucosylceramide
Fabry	#301500	*GLA*	α-Galactosidase A	Globotriaosylceramide
Niemann–Pick	#257200 (type A) #607616 (type B)	*SMPD1*	Acid sphingomyelinase	Sphingomyelin
	#257220 (type C1)	*NPC1*	NPC intracellular cholesterol transporter 1	Cholesterol
	#607625 (type C2)	*NPC2*	NPC intracellular cholesterol transporter 2	
Krabbe	#245200	*GALC*	β-Galactosylceramidase	β-Galactosylsphingosine
Farber lipogranulomatosis	#228000	*ASAH1*	Acid ceramidase	Ceramide
GM1 gangliosidosis	#230500 (type I) #230600 (type II) #230650 (type III)	*GBL1*	β-Galactosidase	GM1 ganglioside
GM2 gangliosidosis	#272750 (AB variant)	*GM2A*	GM2 activator protein	GM2 ganglioside
	#272800 (Tay-Sachs)	*HEXA*	Hexosaminidase α-subunit	
	#268800 (Sandhoff)	*HEXB*	Hexosaminidase β-subunit	
Metachromatic leukodystrophy	#250100	*ARSA*	Arylsulfatase A	Sulfo-galactosylceramide

**Table 2:** Zebrafish models of sphingolipidoses.

Disease	Human Gene	Zebrafish Orthologous	Zebrafish Model
Gaucher	*GBA1*	*gba1*	MO [138] Spontaneous mutation [138] TALEN [139] CRISPR/Cas9 [117,140]
Fabry	*GLA*	*gla*	CRISPR/Cas9 [131]
Niemann–Pick	*SMPD1* *NPC1* *NPC2*	*smpd1* *npc1* *npc2*	CRISPR/Cas9 [122] MO [141] CRISPR/Cas9 [142,143,144,145]
Krabbe	*GALC*	*GALCa* *GALCb*	MO [146]
Farber lipogranulomatosis	*ASAH1*	*asah1a* *asah1b*	MO [147] CRISPR/Cas9 [111]
GM2 gangliosidosis	*HEXA* *HEXB*	*hexa* *hexb*	MO [148,149] CRISPR/Cas9 [150]
Metachromatic leukodystrophy	*ARSA*	*arsa*	MO [148]

## Data Availability

Not applicable.

## Abbreviations

aSMase, acid sphingomyelinase; *CERS*, ceramide synthases; CRISPR/Cas9, clustered regularly interspersed short palindromic repeats/CRISPR-associated 9; dpf, days post fertilization; ER, endoplasmic reticulum; ERT, enzyme replacement therapy; *GALC*, β-galactosylceramidase; Gb3, globotriaosylceramide; GD, Gaucher disease; GM2AP, GM2 activator protein; HEXA, β-hexosaminidase A; HSCT, hematopoietic stem cell transplantation; hpf, hours post fertilization; KD, knockdown; KO, knockout; LC, liquid chromatography; M6P, mannose 6-phosphate; MLD, metachromatic leukodystrophy; MO, morpholino; MS, mass spectrometry; NPD, Niemann–Pick disease; S1P, sphingosine-1-phosphate; S1PR, S1P receptor; *SMS*, sphingomyelin synthase; SRT, substrate reduction therapy; TALEN, transcription activator like effector nuclease; WISH, whole mount in situ hybridization.
